# Comparative study of curcumin and curcumin formulated in a solid dispersion: Evaluation of their antigenotoxic effects

**DOI:** 10.1590/S1415-475738420150046

**Published:** 2015

**Authors:** Leonardo Meneghin Mendonça, Carla da Silva Machado, Cristiane Cardoso Correia Teixeira, Luis Alexandre Pedro de Freitas, Maria Lourdes Pires Bianchi, Lusânia Maria Greggi Antunes

**Affiliations:** 1 Departamento de Análises Clínicas, Toxicológicas e Bromatológicas, Faculdade de Ciências Farmacêuticas de Ribeirão Preto, Universidade de São Paulo, Ribeirão Preto, SP, Brazil.; 2 Departamento Farmacêutico, Universidade Federal de Juiz de Fora, Campus Governador Valadares, Governador Valadares, MG, Brazil.; 3 Departamento de Genética, Faculdade de Medicina de Ribeirão Preto, Universidade de São Paulo, Ribeirão Preto, SP, Brazil.; 4 Departamento de Ciências Farmacêuticas, Faculdade de Ciências Farmacêuticas de Ribeirão Preto, Universidade de São Paulo, Ribeirão Preto, SP, Brazil.

**Keywords:** Curcuma longa, antigenotoxicity, micronucleus test, DNA damage, comet assay

## Abstract

Curcumin (CMN) is the principal active component derived from the rhizome of *Curcuma longa* (*Curcuma longa* L.). It is a liposoluble polyphenolic compound that possesses great therapeutic potential. Its clinical application is, however, limited by the low concentrations detected following oral administration. One key strategy for improving the solubility and bioavailability of poorly water-soluble drugs is solid dispersion, though it is not known whether this technique might influence the pharmacological effects of CMN. Thus, in this study, we aimed to evaluate the antioxidant and antigenotoxic effects of CMN formulated in a solid dispersion (CMN SD) compared to unmodified CMN delivered to Wistar rats. Cisplatin (cDDP) was used as the damage-inducing agent in these evaluations. The comet assay results showed that CMN SD was not able to reduce the formation of cDDP-DNA crosslinks, but it decreased the formation of micronuclei induced by cDDP and attenuated cDDP-induced oxidative stress. Furthermore, at a dose of 50 mg/kg b.w. both CMN SD and unmodified CMN increased the expression of *Tp53* mRNA. Our results showed that CMN SD did not alter the antigenotoxic effects observed for unmodified CMN and showed effects similar to those of unmodified CMN for all of the parameters evaluated. In conclusion, CMN SD maintained the protective effects of unmodified CMN with the advantage of being chemically water soluble, with maximization of absorption in the gastrointestinal tract. Thus, the optimization of the physical and chemical properties of CMN SD may increase the potential for the therapeutic use of curcumin.

## Introduction

Curcumin (1,7-bis[4-hydroxy 3-methoxy phenyl]-1,6-heptadiene-3,5-dione, CMN) is the principal active component derived from the rhizome of *Curcuma longa* (*Curcuma longa* L.), which is commonly used in Ayurvedic and Chinese medicine, and serves in numerous other countries as a coloring agent or spice in many food preparations (Goel *et al*., 2008). CMN is a liposoluble polyphenolic compound, structurally consisting of two ring methoxyphenols attached to a β-diketone structure. The phenolic groups and β-diketone are structures that are characteristic of antioxidant compounds and are critical for the antioxidant action of CMN (Singh *et al*., 2011).

CMN possesses an antioxidant capacity similar to that of potent antioxidants, such as the vitamin E analog trolox (Somparn *et al*., 2007). Studies have suggested that CMN inhibits lipid peroxidation in different tissues (Sreejayan and Rao, 1994), acts as an effective scavenger of intracellular reactive oxygen species (ROS) (Barzegar and Moosavi-Movahedi, 2011), and regulates intracellular levels of antioxidant enzymes (Naik*et al*., 2004). In addition to its recognized antioxidant activity, CMN possesses other pharmacological activities, including anti-inflammatory, anticancer and antidepressant properties (Aggarwal *et al*., 2013; Esatbeyoglu *et al*., 2015), and has been described as an antigenotoxic and antitumoral agent (Mendonça *et al*., 2009).

CMN also exhibits antigenotoxic effects in *in vivo* and *in vitro* models via reducing the chromosomal damage induced by physical and chemical agents (Antunes *et al*., 1999; Mendonça *et al*., 2009). The antioxidant and free-radical scavenging properties of CMN are considered important factors in its role in maintaining genomic stability, as oxidative stress can modify nitrogenous bases and result in DNA strand breaks (Premkumar *et al*., 2004).

Other biological effects of CMN include induction of cell cycle arrest, inhibition of cell proliferation, induction of apoptosis and modulation of gene expression (Zhou *et al*., 2011). In addition to acting at different levels of regulation of the process of cell growth and apoptosis, CMN operates in the initial processes of carcinogenesis by controlling chromosomal alterations and DNA damage (Duvoix *et al*., 2005).

Although CMN exhibits great therapeutic potential, its clinical application is frequently limited by the low blood concentrations obtained following oral administration. The low oral bioavailability of CMN was first demonstrated by Wahlstrom and Blennow (1978) and was attributed to poor absorption in the gastrointestinal tract, rapid metabolism, and rapid systemic elimination. Thus, studies have been performed with the aim of increasing the bioavailability of CMN. These involved synthesized analogues, combined use with CMN metabolism inhibitors (such as piperin) or newly developed formulations, such as nanoparticles, micelles, phospholipid complexes, and solid dispersions (Aggarwal and Harikumar, 2009).

Solid dispersion of drugs is an important strategy for improving the solubility of poorly water-soluble compounds, which often display low oral bioavailability, as is the case with CMN (Seo *et al*., 2012). This technology mixes one or more pharmacologically active compounds on a carrier, with the goal of altering their physicochemical properties, such as their stability, solubility and dissolution rate, which may result in greater bioavailability (Vasconcelos *et al*., 2007).

The evaluation of early genotoxicity is an essential part of the regulatory requirement and welfare considerations. In this study, we performed an *in vivo* comparative analysis between CMN formulated in a ternary solid dispersion (SD) composed of curcumin/gelucire^®^50-13/aerosil^®^ (CMN SD) and unmodified CMN to assess whether CMN SD can induce chromosomal damage or interfere with the recognized antioxidant and antigenotoxic properties of unmodified CMN. For this purpose, we measured genomic damage by means of comet assay in kidney and peripheral blood cells, as well as the micronucleus test in bone marrow from rats. We also evaluated oxidative stress via the analyses of reduced glutathione (GSH) and thiobarbituric-acid-reactive substances (TBARS) and examined the expression of *Tp53* mRNA in the kidney tissue.

## Materials and Methods

### Chemicals

CMN for CMN SD formulation was purchased from Asian Herbex Ltd (Hyderabad, India), gelucire^®^ 50/13 was gently donated by Gattefosse Corp (Saint-Priest, France) and aerosil^®^ obtained from EvonikInd AG (Germany). Unmodified CMN (CAS 458-37-7) was purchased from Sigma-Aldrich (St. Louis, MO, USA). The mixture of gelucire^®^50-13/aerosil^®^ (GLA), which were components used in the preparation of CMN SD, was employed as a control in these experiments at a concentration equivalent to the highest applied dose of CMN SD.

Cisplatin (cDDP), which was used as a damage-inducing agent due to its recognized genotoxic and nephrotoxic effects (Antunes *et al*., 2001), was purchased from Quiral Química do Brasil (Platinil^®^, Juiz de Fora, Brazil). Trypan Blue (CAS 72-57-1), ethylenediaminetetraacetic acid (EDTA, CAS 60-00-4), Triton X-100 (CAS 9002-93-1) and Tris (CAS 77-86-1) were obtained from Sigma-Aldrich (St. Louis, MO, USA). Low melting point agarose (CAS: 9012-36-6) and normal melting point agarose (CAS: 9012-36-6) were purchased from Invitrogen (California, CA, USA). Dimethylsulfoxide was obtained from Merck (Darmstadt, Hessen, Germany). Other reagents were of analytical grade and of the purest quality available.

### Preparation of the solid dispersion

CMN SD was prepared by the spray drying method. The carrier, Gelucire® 50/13 (Gatefosse, France), was melted in a water bath, and a solution of CMN in 50% ethanol was added (GLC: CUR, 1:1). This suspension containing equal parts of CMN and carrier was homogenised with a high shear mixer at 18,000 rpm and Aerosil (EvonikInd AG, Germany) was slowly added until 20% (w/w). Further homogenisation using a high shear mixer (14,000 rpm) was performed for 7 min. The suspension obtained by this procedure was dried in a lab-scale spray dryer model MSD 0.5 (Labmaq Ltd., Ribeirão Preto, Brazil) using the following set conditions: suspension feed rate of 5 mL/min, atomisation air pressure of 4 kgf/cm^2^, drying air rate of 1.5 m^3^/min, air outlet temperature of 40 °C and a suspension solids content of 7.5% (w/w).

### Characterisation and stability of CMN SD

The CMN SD microparticles were characterised by particle size, water activity, CMN content and solubility. Additionally, CMN SD physical-chemical properties were characterised by scanning electron microscopy (SEM), differential scanning calorimetry (DSC), thermogravimetry (TGA), infrared spectroscopy (FTIR) and X-ray powder diffraction (XRPD). Stability was assessed by DSC, TG, XRPD and FTIR after 3 and 6 months for samples kept at room temperature (25 °C) in triplicate. The stability was also evaluated by observing the solubility of samples after 3, 6 and 9 months of storage at room temperature. The CMN SD microparticles resulted in a mean diameter of 550 mm, and CMN content of 338.4 mg/g. The thermal analysis by DSC and TGA showed no interaction among the components of CMN SD and this result was confirmed by the observations from FTIR and XRPD. The same was observed for these solid state characteristics after 3, 6 and 9 months, demonstrating an excellent stability of the microparticles. CMN solubility in its CMN SD form was determined to be 2.7 μg/mL. Studies suggested that CMN SD is approximately 6.75 fold more water-soluble in comparison to unmodified CMN (Yallapu *et al*., 2012). The *in vitro* dissolution profiles of CMN-SD in phosphate buffer pH 7.4 revealed that the release was 80% in only 10 min.

### Animals

Male Wistar albino rats, at 5-6 weeks of age and weighing approximately 160 g were obtained from the Animal Facility of the Ribeirão Preto Campus of the University of São Paulo. The animals were divided into 12 groups of six for each treatment. The experimental protocols applied in this study were approved by the Local Ethics Committee for Animal Use (CEUA) of Ribeirão Preto, Brazil, Register No.08.1.1417.53.2.

The rats were maintained in polypropylene cages with steel wire tops (three per cage), and the environmental controls were set to maintain conditions of 22 ± 2 °C and 55 ± 10% relative humidity under a 12-h light-dark cycle. Fresh water and food were provided *ad libitum*. This study complied with national and international laws, and it was conducted in accordance with the conditions for animal care recommended by the Canadian Council on Animal Care (Olfert and McWilliam, 1993).

### Experimental design

To determine whether the well-established protective effect of unmodified CMN demonstrated in other studies (Antunes *et al*., 1999) was also observed for CMN SD, treatments were performed with CMN SD (at 5, 25 and 50 mg/kg b.w.), unmodified CMN at 50 mg/kg b.w., saline solution or GLA. These were administered via gavage at 72 h, 48 h, 24h or 30 min before the intraperitoneal administration of saline solution or the antitumoral agent cDDP, which was used as a damage-inducing agent.

The body weights of the rats were recorded daily. At 24 h after cDDP administration (5^th^ day), the animals were euthanized for sample collection. The dose of unmodified CMN applied in this study was defined from previously published studies in rodents (Ganta *et al*., 2010; Yu *et al*., 2011) and due the absence of toxic effects at macroscopic levels; and the dose of cDDP (6 mg/kg b.w.) was selected based on other studies that have shown that this dose induces chromosomal damage in rodents (Serpeloni *et al*., 2013). Adequate mass/mass relationship of CMN in unmodified CMN and CMN SD preparations were taken into consideration to obtain the doses used in the experiments. The same animals were used in genotoxicity assays (micronucleus test and comet assay) and biochemical tests (GSH and TBARS), as well as for the expression analysis of the *Tp53* gene.

### Alkaline comet assay

The alkaline version of the comet assay was performed according to protocols proposed by Singh *et al*. (1988) and Tice *et al*. (2000), with minor modifications (the slides were stained with GelRed, 1:10,000, Biotium-USA). To check for possible cytotoxic effects of the treatments, cell viability was determined via the Trypan blue dye exclusion method. Samples of peripheral blood and kidney cell suspensions (0.2 g of kidney tissue sliced into fragments in a Petri dish containing 2 mL of chilled Hank’s solution) were mixed with 0.5% low melting point agarose dissolved in phosphate buffered saline and spread on microscope slides precoated with 1.5% normal melting agarose. The slides were immersed in freshly prepared lysis solution consisting of 2.5 M NaCl, 100 mM EDTA, 1% Triton X-100, and 10 mMTris (pH 10) for at least 24 h at 4 °C. Following lysis, the slides were placed in a horizontal electrophoresis unit containing 300 mM NaOH and 1 mM EDTA (pH > 13) for 20 min at an electric field strength of 0.78 V/cm (25 V and 300 mA). The slides were neutralized and stained with Gel Red (1:10,000). A total of 100 nucleoids per animal (two slides of 50 nucleoids each) were analysed at a 400x magnification using a fluorescence microscope (Axiostar, Zeiss, Germany) equipped with a 515-560 nm excitation filter, a 590 nm barrier filter and an integrated digital camera. Tail intensity (% tail DNA) was evaluated using the Comet Assay IV software (Perceptive Instruments, Suffolk, UK).

### Micronucleus test

The micronucleus test was performed according to the protocol described by Schmid (1975). Bone marrow cells were harvested from rat femurs, mixed with fetal bovine serum, homogenized and centrifuged, and the pellet was resuspended for slide preparations. The slides were then fixed, stained with Giemsa solution and coded. Three slides were produced for each animal. Coded slides were scored under 1000X magnification using a light microscope (Zeiss). For each of the six animals per group, 2000 polychromatic erythrocytes (PCEs) were scored, and the number of micronucleated PCE (MNPCE) was recorded. The percentage of PCE among 500 erythrocytes was calculated as a measure of erythroblast proliferation [PCE/(PCE + NCE)].

### TBARS and GSH levels in the kidney

TBARS measurements in kidney tissue were performed according to Buege and Aust (1978). A 0.5 mL aliquot of the homogenate was added to 1 mL of thiobarbituric acid solution (containing 15% trichloroacetic acid and 0.25 M HCl) to a final concentration of 26 nM. This mixture was warmed in a water bath for 15 min and centrifuged for 20 min at 180 x *g*. The absorbance of the supernatant was determined at 535 nm (UV-VisB582 Micronal spectrophotometer), and the results were expressed as nmol TBARS/mg protein. The breakdown of the product 1,1,3,3-tetraetoxypropane was used as the standard reaction.

GSH concentrations in kidney tissue were determined according to method described by Sedlak and Lindsay (1968). The homogenate samples were diluted in water (1:4), precipitated with 50% trichloroacetic acid and then centrifuged at 150 x *g* for 10 min. A 2.0 mL volume of Tris-EDTA buffer (0.2 M, pH 8.9) and 0.1 mL of 5,5 ‘-dithio-bis-2-nitrobenzoic acid (DTNB) in 0.01 M methanol were added to a 0.5 mL aliquot of the supernatant. The samples were maintained at room temperature for 15 min and then read at 412 nm (RayLeigh UV-1601 spectrophotometer). Standard curves were prepared using α-cysteine, and results were expressed as nmol GSH/g protein.

The quantification of total proteins was done at 650 nm (RayLeigh UV-1601 spectrophotometer) using Lowry’s method (Hartree, 1972).

### Quantification of *Tp53* mRNA

Total RNA was extracted from kidney tissue using the SV Total Isolation System kit (Promega, Madison, WI, USA), according the manufacturer’s instructions. The integrity of the extracted RNA was assessed via gel electrophoresis in 1.0% agarose, and the purity was measured based on the ratios of the spectrophotometric optical density measurements taken at 260 nm/280 nm and 260 nm/230 nm. The extracted RNA was converted to cDNA using the SuperScriptTM III kit (Invitrogen, Carlsbad, CA, USA), and RT-qPCR was performed in a CFX96 Real-Time PCR Detection System (Bio-Rad, CA, USA) using the Bio-Rad Real-Time PCR system with ABsoluteTM QPCR SYBR1 Green Mix (Invitrogen, Carlsbad, CA, USA), where fluorescence detection was performed following each annealing/extension cycle.

The following reference genes were tested for suitability: b-actin (b-actin-forward: TCCTGTGGCATCCAT GAACT; b-actin reverse: CCAGGGCAGTAATCTCTTT CTTCTG), GAPDH (GAPDH-forward: GGCATCGTGG AAGGGCTCAT; GAPDH-reverse: GCCATCACGCC ACAGCTTTC) and HKI (HKI-forward: GCGAGGGGA CTATGATGCT; HKI-reverse CGCAGTTCCTCCATGT AGC). Based on stability, we selected b-actin as the endogenous control gene for RT-qPCR. Gene-specific primers for *Tp53* (*Tp53*-forward: CATCATCACGCTGGAAGAC TC; *Tp53*-reverse: TTCAGCTCTCGGAACATCTC) and b-actin (Nair *et al*., 2004) were synthesized by Sigma-Aldrich (St. Louis, MO, USA).

RT-qPCR efficiencies for *Tp53* and b-actin were satisfactory, and the relative expression of *Tp53* mRNA was normalized to the amount of b-actin using the method of relative 2^−ΔΔCt^ quantification described by Livak and Schmittgen (2001).

### Data analysis

Statistical analysis was performed using GraphPad Prism 5.0 software. The results are expressed as the means ± standard deviation. Analysis of variance (ANOVA) followed by Tukey’s *post hoc* tests was employed to calculate statistically significant differences (at p < 0.05) in the results obtained for the treatment *vs*. saline solution group.

## Results

### Variation in body mass and the relative mass of the kidney

Body weights of the animals were recorded daily (Table 1. Prior to the intraperitoneal injection cDDP, no variation in body weight gain was observed in any group. The experimental groups that received cDDP intraperitoneally showed reduced body weight gain compared to the saline control group. Combined application of CMN SD or unmodified CMN with cDDP did not alter the reduction of body weight gain triggered by cDDP. We measured the kidney weight/body weight ratio as a toxicity parameter. No difference was observed between the treatment groups and the saline solution group for this parameter (Table 1).

**Table 1:** Evaluation of the variation of mass gain in rats after subacute treatment with CMN SD, unmodified CMN, cDDP and their associations.

Treatments	Body weight (g)^a^ mean ± standard deviation	Body weight (g)^b^ mean ± standard deviation	Kidney/body weight (%)
Saline solution	29.7 ± 6.4	11.3 ± 2.1	0.98 ± 0.03
GLA	31.3 ± 5.6	9.3 ± 4.7	1.01 ± 0.04
CMN SD 5	31.2 ± 6.6	11.0 ± 2.8	0.93 ± 0.05
CMN SD 25	28.0 ± 4.6	9.2 ± 7.3	0.93 ± 0.09
CMN SD 50	25.5 ± 8.2	8.5 ± 1.0	1.00 ± 0.11
CMN 50	31.7 ± 6.6	8.2 ± 2.4	0.96 ± 0.05
cDDP	34.0 ± 4.3	0.6 ± 3.9*	1.03 ± 0.04
GLA + cDDP	27.5 ± 6.1	0.8 ± 2.2*	1.02 ± 0.11
CMN SD 5 + cDDP	29.7 ± 3.8	1.8 ± 1.5*	0.97 ± 0.05
CMN SD 25 + cDDP	26.7 ± 5.8	2.3 ± 4.8*	0.97 ± 0.06
CMN SD 50 + cDDP	23.0 ± 5.3	0.2 ± 4.5*	1.00 ± 0.09
CMN 50 + cDDP	31.8 ± 4.7	0.1 ± 2.5*	0.90 ± 0.06

cDDP: cisplatin (6 mg/kg b.w.); CMN: curcumin (5, 25 and 50 mg/kg b.w.); GLA: gelucire®50-13/aerosil®; SD: solid dispersion.

a Interval 1- variation in body mass, in grams (g), between days 1 and 4 of the experimental period.

b Interval 2 -variation in body mass, in grams (g), between day 4 and 5 of the experimental period. The results represent the mean ± standard deviation for each group (six animals/group).

* Significantly different from saline solution group, assessed by ANOVA and Tukey’s *post hoc* test (p < 0.05).

### CMN SD reduces chromosomal damage induced by cDDP

The capacity of CMN SD or unmodified CMN to reduce DNA and chromosomal damage induced by cDDP was evaluated using the comet and micronucleus assays, respectively.

Cell viability observed in the kidney and peripheral blood was greater than 70% in all of the analysed groups, in accordance with recommendations for performing a comet assay analysis (Azqueta and Collins, 2013), as shown in Table 2. In the comet assay, the extent of DNA damage was assessed based on the tail intensity parameter (% tail DNA).

**Table 2:** Tail Intensity (% tail DNA) and cell viability (expressed as % in relation to saline solution group) in cells of kidney and peripheral blood after subacute treatment with CMN SD, unmodified CMN, cDDP and their associations, analyzed in the comet assay.

Treatments	% tail DNA		Cell viability (%)	
	Kidney	Peripheral blood	Kidney	Peripheral blood
Saline solution	7.4 ± 4.0	4.4 ± 2.1	100.0 ± 0.0	100.0 ± 0.0
GLA	8.3 ± 2.9	2.6 ± 0.7	88.5 ± 1.9	98.8 ± 0.7
CMN SD 5	9.4 ± 2.8	4.7 ± 1.6	91.2 ± 2.4	99.2 ± 0.7
CMN SD 25	6.7 ± 1.7	4.8 ± 0.6	88.0 ± 2.6	98.7 ± 0.5
CMN SD 50	7.5 ± 3.0	2.1 ± 1.1	88.5 ± 3.3	99.0 ± 0.6
CMN 50	5.5 ± 2.8	3.2 ± 0.9	89.0 ± 2.3	98.7 ± 0.5
cDDP	3.4 ± 1.1*	2.5 ± 0.4	89.7 ± 1.7	99.2 ± 0.7
GLA + cDDP	3.8 ± 0.7*	2.2 ± 0.8	86.5 ± 3.1	98.8 ± 0.7
CMN SD 5 + cDDP	5.0 ± 1.1	3.0 ± 1.7	90.5 ± 2.5	99.8 ± 0.7
CMN SD 25 + cDDP	6.4 ± 1.1	4.4 ± 1.2	87.3 ± 3.6	98.5 ± 1.2
CMN SD 50 + cDDP	5.7 ± 1.1	3.3 ± 1.5	85.5 ± 4.4	98.5 ± 1.4
CMN 50 + cDDP	4.0 ± 0.8	2.1 ± 0.6	88.5 ± 2.2	99.2 ± 0.7

Saline solution or cDDP was administered intraperitoneally 30 min after the last gavage of CMN SD or unmodified CMN. %: percentage; cDDP: cisplatin (6 mg/kg b.w.); CMN: curcumin; GLA: gelucire®50-13/aerosil®; SD: solid dispersion. The results represent the mean ± standard deviation for each group (six animals/group).

* Significantly different from saline solution group, assessed by ANOVA and Tukey’s *post hoc* test (p < 0.05).

No genotoxic effects of CMN SD, unmodified CMN or GLA were observed in kidney or peripheral blood cells (Table 2). The results regarding % tail DNA observed in the animals treated with cDDP revealed a significant decrease in DNA migration compared to the saline solution group in renal tissue, but not in peripheral blood (Table 2). The results for the cDDP group indicated the formation of crosslinks with DNA. Treatment with CMN SD or unmodified CMN in association with cDDP did not induce significant alterations compared to the cDDP-only group in the comet assay.

The PCE/(NCE + PCE) ratio revealed no significant differences between the treatment groups and saline solution group (Table 3), indicating that none of the treatments altered the rate of cell division in bone marrow. Table 3 shows the effect of CMN SD or unmodified CMN, either combined with cDDP or not, on the formation of micronuclei. CMN SD, unmodified CMN and the GLA mixture did not induce micronucleus formation. In contrast, cDDP treatment significantly increased the frequency of MNPCE compared to the saline solution group. CMN SD (at 5, 25 and 50 mg/kg b.w.) and unmodified CMN (50 mg/kg b.w.) significantly reduced the formation of cDDP-induced micronuclei (p < 0.05). This effect occurred to a similar extent under treatment with CMN SD and CMN at a dose of 50 mg/mL.

**Table 3:** Frequency of micronucleated polychromatic erythrocytes (MNPCE) and the percentage (%) of PCE/(PCE + NCE) in 500 erythrocytes in the bone morrow of Wistar rats treated with CMN SD, unmodified CMN, cDDP or their associations, analyzed in the micronucleus test.

Treatments	Bone marrow erythrocytes	
	MNPCEs/1000 PCEs	PCE/(PCE + NCE) ratio (%)
Saline solution	1.25 ± 0.78	52.6 ± 4.6
GLA	1.92 ± 1.1	54.7 ± 6.5
CMN SD 5	2.25 ± 1.13	59.3 ± 6.2
CMN SD 25	1.5 ± 0.82	53.4 ± 10.4
CMN SD 50	2.42 ± 1.09	43.2 ± 6.0
CMN 50	1.91 ± 1.27	55.7 ± 8.8
cDDP	13.25 ± 3.51*	49.9 ± 5.6
GLA + cDDP	12.75 ± 3.32*	44.8 ± 4.9
CMN SD 5 + cDDP	6.88 ± 1.77* #	51.8 ± 5.9
CMN SD 25 + cDDP	5.33 ± 2.98* #	53.9 ± 8.3
CMN SD 50 + cDDP	6.42 ± 3.12* #	51.1 ± 3.0
CMN 50 + cDDP	7.08 ± 3.04* #	51.0 ± 7.5

Saline solution or cDDP was administered intraperitoneally 30 min after the last gavage of CMN SD or unmodified CMN. cDDP: cisplatin (6 mg/kg b.w.); CMN: curcumin (5, 25 and 50 mg/kg b.w.); GLA: gelucire®50-13/aerosil®; SD: solid dispersion. The results represent the mean ± standard deviation for each group (six animals/group).

* Significantly different from saline solution group.

# Significantly different from cDDP group, assessed by ANOVA and Tukey’s *post hoc* test (p < 0.05).

### CMN SD attenuates cDDP-induced oxidative stress

Oxidative stress was evaluated by measuring the concentrations of TBARS and GSH in renal tissue 24 hours after cDDP administration. When administered alone, CMN SD and unmodified CMN did not alter the GSH and TBARS concentrations detected in renal tissue (Table 4). cDDP significantly increased TBARS levels compared to the saline solution group but did not alter GSH levels. CMN SD or unmodified CMN, administered together with cDDP, was able to maintain the TBARS levels observed in the saline solution group (p >; 0.05) (Table 4). There was no significant difference between the groups treated with GLA together with cDDP *vs*. cDDP alone.

**Table 4:** Evaluation of reduced glutathione (GSH) and thiobarbituric-acid-reactive substances (TBARS) in the kidney of Wistar rats treated with CMN SD, unmodified CMN, cDDP or their associations.

Treatments	GSH (nmol/mg protein)	TBARS(nmol/mg protein)
Saline solution	18.9 ± 0.4	0.249 ± 0.011
GLA	17.7 ± 2.6	0.226 ± 0.023
CMN SD 5	14.2 ± 1.3	0.237 ± 0.018
CMN SD 25	15.3 ± 1.1	0.232 ± 0.026
CMN SD 50	17.2 ± 1.7	0.247 ± 0.018
CMN 50	14.4 ± 1.2	0.236 ± 0.018
cDDP	15.2 ± 3.5	0.302 ± 0.026*
GLA + cDDP	18.5 ± 0.6	0.286 ± 0.014*
CMN SD 5 + cDDP	16.6 ± 1.3	0.215 ± 0.009#
CMN SD 25 + cDDP	14.0 ± 1.7	0.200 ± 0.011#
CMN SD 50 + cDDP	19.1 ± 2.5* #	0.218 ± 0.018#
CMN 50 + cDDP	17.0 ± 1.4	0.222 ± 0.064#

Saline solution or cDDP was administered intraperitoneally 30 min after the last gavage of CMN SD or unmodified CMN. cDDP: cisplatin (6 mg/kg b.w.); CMN: curcumin (5, 25 and 50 mg/kg b.w.); GLA: gelucire®50-13/aerosil®; SD: solid dispersion. The results represent the mean ± standard deviation for each group (six animals/group).

* Significantly different from saline solution and GLA groups.

# Significantly different from cDDP group, assessed by ANOVA and Tukey’s *post hoc* test (p < 0.05).

### *Tp53* mRNA levels are affected by CMN SD

**Figure 1:**
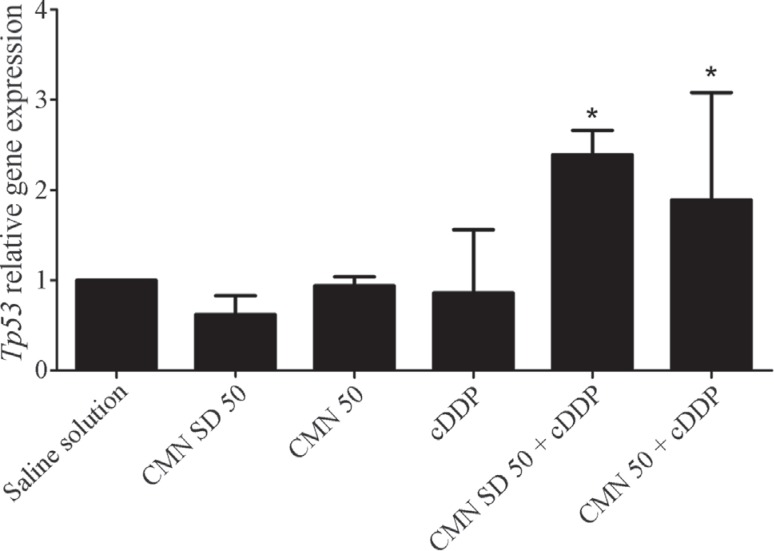
Relative quantification of *Tp53* mRNA in kidney cells following treatment with CMN SD (50 mg/kg b.w.), unmodified CMN (50 mg/kg b.w.) and cDDP alone or in combination. The housekeeping gene b-actin was used for normalization of the samples. The results represent the mean ± standard deviation for each group (six animals/group). *Statistically significantly different from the saline solution group, as assessed by ANOVA and Tukey’s *post hoc* test (p < 0.05).

Figure 1 shows the effects of CMN SD (50 mg/kg b.w.) and unmodified CMN (50 mg/kg b.w.), either alone or in association with cDDP (6 mg/kg b.w.), on the *Tp53* mRNA levels in kidney tissue. The results showed that CMN SD, unmodified CMN and cDDP did not alter *Tp53* gene expression compared to the saline solution group. However, when either CMN SD or unmodified CMN was administered together with cDDP, *Tp53* expression was up-regulated compared to saline solution group. There was no difference in the levels of *Tp53* mRNA in kidney cells when comparing the CMN SD and unmodified CMN groups.

## Discussion

With the objective of evaluating whether CMN SD could induce chromosomal damage or interfere with the recognized antioxidant and antigenotoxic properties of unmodified CMN, we performed an *in vivo* comparative analysis between CMN SD and unmodified CMN, by measuring DNA damage, evaluating oxidative stress and analysing *Tp53* mRNA levels. Our results showed that CMN SD decreased chromosomal damage induced by cDDP, up-regulated *Tp53* expression when administered together with cDDP, and attenuated cDDP-induced oxidative stress. There were no significant differences observed between the effects of CMN SD and unmodified CMN for any of the parameters evaluated in this study.

cDDP was used as the damage-inducing agent in this study due its genotoxic and nephrotoxic effects, and the kidney was evaluated as a target organ. The genotoxic mechanisms of cDDP involve chromosomal damage, as demonstrated by the induction of micronuclei (Gupta *et al*., 2011); the formation of cDDP-DNA crosslinks, as shown by the decrease in the percentage of DNA in the tail (Stang and Witte, 2009), and the regulation of *Tp53* mRNA and p53 protein levels (Yuan *et al*., 2011). The *in vivo* mechanisms of cDDP nephrotoxicity are mainly related to the induction of oxidative stress: cDDP increases free radical production (Ognjanovic *et al*., 2012) and decreases antioxidant enzyme activity (Badary *et al*., 2005).

According to Wolfsegger *et al*. (2009), the changes in total mass of an animal and the relationship between organ weights and total mass of an animal can be used as an indication of the toxicity of a compound under evaluation. Animals treated with CMN SD and unmodified CMN showed no difference in total mass compared to the saline solution group. However, there was a significant reduction in body mass of the rats treated with cDDP (6 mg/kg b.w.) compared to the saline solution group. Other studies in rodents have also shown a decrease in body mass following cDDP administration at the same dose as applied in this study (Zhang *et al*., 1999). This is most likely due to cytotoxic effects of cDDP.

Assessment of chromosomal damage was performed via the micronucleus test in erythrocytes from bone marrow, and DNA damage was evaluated using the comet assay in kidney and peripheral blood samples. These two tests are frequently employed to evaluate the genotoxic and mutagenic effects of physical or chemical agents, where by the comet assay can detect initial lesions in DNA, and the micronucleus assay can detect chromosomal breaks and losses (Bowen *et al*., 2011; Collins, 2015). DNA lesions detected by the comet assay can be single- and double-strand breaks, alkaline-labile sites and DNA-DNA and DNA-protein crosslinks. Single- or double-strand breaks and alkaline-labile sites are further identified in the comet assay as an increase in DNA migration, while DNA-DNA and DNA-protein crosslinks are detected as a decrease in DNA migration (Nesslany *et al*., 2007).

CMN SD did not induce DNA or chromosomal damage in any of the analysed tissues, suggesting that CMN SD did not induce genotoxicity in these. Regarding the antigenotoxicity action of CMN SD, we saw that it reduced micronucleus formation in the bone marrow cells of rats exposed to cDDP, but did not reduce the formation of cDDP-DNA crosslinks observed in kidney. The antigenotoxicity of CMN SD was similar to that of unmodified CMN in this study, a finding that is comparable to that reported in other studies involving CMN (Mendonça *et al*., 2009; Celik *et al*., 2013). These results furthermore suggest that the protective mechanism of CMN SD is not related to a reduction in the formation of cDDP-DNA crosslinks, since CMN SD did not interfere with the mechanism of cDDP genotoxicity. It seems, however, related with the reduction of cDDP-induced breaks and loss of chromosomes.

Various studies have demonstrated the relevance of oxidative stress in cDDP-induced cellular damage. Oxidative stress can cause DNA damage, resulting in strand breaks, alterations in gene expression, and mutations (Cooke *et al*., 2003). Some antioxidant agents may exert their protective effects by increasing the capacity of cellular antioxidant defense systems, or via the sequestration of reactive species (Costa *et al*., 2012), and the protective effects of CMN, as well as its antigenotoxicity activity are often related to its antioxidant properties.

In this study, the evaluated oxidative stress parameters were the GSH concentration and TBARS formation in renal tissue. It is generally accepted that the mechanism by which cDDP induces oxidative stress in renal tissue involves the induction of lipid peroxidation (Ognjanovic *et al*., 2012), and the antioxidant properties of CMN are related to its ability to modulate the concentrations of GSH and TBARS (Biswas *et al*., 2005; Kaur *et al*., 2006). Some findings suggest that CMN could be useful in reducing the nephrotoxicity of cDDP (Swamy *et al*., 2012), and our results showed that CMN SD, when administered together with cDDP, was able to maintain the TBARS levels observed in the saline solution group. These results suggest that CMN SD, processed via spray dry technology, can protect against cDDP-induced lipid peroxidation in the kidney and maintain TBARS at basal levels.

In addition, we assessed the expression of *Tp53* in kidney cells because the involvement of p53 protein has been implicated in cDDP toxicity in normal cells, as observed in nephrotoxicity (Jiang and Dong, 2008). Like unmodified CMN, CMN SD increased the mRNA levels of *Tp53* when administered together with cDDP in renal tissue, compared to the saline group; and they did not alter the *Tp53* mRNA levels when compared to the cDDP group. In renal tubule cells, p53 proteins inhibitors are thought to interfere with the efficacy of cDDP (Jiang and Dong, 2008). While the results obtained in the present study suggest that CMN SD did not interfere with cDDP in *Tp53* gene expression, it was not possible to rule out effects on p53 protein.

Recent studies have suggested an “integrated toxicology” strategy to define the pharmacological and biological potential of new compounds, and genotoxicity assays have been of great relevance for the development of new drugs (Hornberg *et al*., 2014). Our findings demonstrate that the technique of producing a solid dispersion containing CMN did not affect the antigenotoxic effects of this compound, and CMN SD showed effects similar to those of unmodified CMN for all of the evaluated parameters. In conclusion, CMN SD maintained the protective and antioxidant effects of unmodified CMN with the advantage of being chemically water soluble. Thus, the optimization of the physical and chemical properties of CMN SD may increase its potential for therapeutic use.

## References

[B1] Aggarwal BB, Harikumar KB (2009). Potential therapeutic effects of curcumin, the anti-inflammatory
agent, against neurodegenerative, cardiovascular, pulmonary, metabolic, autoimmune
and neoplastic diseases. Int J Biochem Cell Biol.

[B2] Aggarwal BB, Yuan W, Li S, Gupta SC (2013). Curcumin-free turmeric exhibits anti-inflammatory and anticancer
activities: Identification of novel components of turmeric. Mol Nutr Food Res.

[B3] Antunes LM, Araujo MC, Dias FL, Takahashi CS (1999). Modulatory effects of curcumin on the chromosomal damage induced by
doxorubicin in Chinese hamster ovary cells. Teratog Carcinog Mutagen.

[B4] Antunes LM, Darin JD, Bianchi MdL (2001). Effects of the antioxidants curcumin or selenium on cisplatin-induced
nephrotoxicity and lipid peroxidation in rats. Pharmacol Res.

[B5] Azqueta A, Collins AR (2013). The essential comet assay: A comprehensive guide to measuring DNA
damage and repair. Arch Toxicol.

[B6] Badary OA, Abdel-Macsoud S, Ahmed WA, Owieda GH (2005). Naringenin attenuates cisplatin nephrotoxicity in rats. Life Sci.

[B7] Barzegar A, Moosavi-Movahedi AA (2011). Intracellular ROS protection efficiency and free radical-scavenging
activity of curcumin. PLoS One.

[B8] Biswas SK, McClure D, Jimenez LA, Megson IL, Rahman I (2005). Curcumin induces glutathione biosynthesis and inhibits NF-kappaB
activation and interleukin-8 release in alveolar epithelial cells: Mechanism of
free radical scavenging activity. Antioxid Redox Signal.

[B9] Bowen DE, Witwell JH, Lillford L, Henderson D, Kidd D, Mc Garry S, Pearce G, Beevers C, Kirkland DJ (2011). Evaluation of a multi-endpoint assay in rats, combining the
bone-marrow micronucleus test, the Comet assay and the flow-cytometric peripheral
blood micronucleus test. Mutat Res.

[B10] Buege JA, Aust SD (1978). Microsomal lipid peroxidation. Methods Enzymol.

[B11] Celik A, Eke D, Ekinci SY, Yildirim S (2013). The protective role of curcumin on perfluorooctane sulfonate-induced
genotoxicity: Single cell gel electrophoresis and micronucleus
test. Food Chem Toxicol.

[B12] Collins AR (2015). The comet assay: A heavenly method!. Mutagenesis.

[B13] Cooke MS, Evans MD, Dizdaroglu M, Lunec J (2003). Oxidative DNA damage: Mechanisms, mutation, and
disease. FASEB J.

[B14] Costa LA, Badawi A, El-Sohemy A (2012). Nutrigenetics and modulation of oxidative stress. Ann Nutr Metab.

[B15] Duvoix A, Blasius R, Delhalle S, Schenekenburger M, Morceau F, Henry E, Dicato M, Diederich M (2005). Chemopreventive and therapeutic effects of curcumin. Cancer Lett.

[B16] Esatbeyoglu T, Ulbrich K, Rehberg C, Rohn S, Rimbach G (2015). Thermal stability, antioxidant, and anti-inflammatory activity of
curcumin and its degradation product 4-vinyl guaiacol. Food Funct.

[B17] Ganta S, Devalapally H, Amiji M (2010). Curcumin enhances oral bioavailability and anti-tumor therapeutic
efficacy of paclitaxel upon administration in nanoemulsion
formulation. J Pharm Sci.

[B18] Goel A, Kunnumakkara AB, Aggarwal BB (2008). Curcumin as “Curecumin”: From kitchen to clinic. Biochem Pharmacol.

[B19] Gupta V, Agrawal RC, Trivedi P (2011). Reduction in cisplatin genotoxicity (micronucleus formation) in non
target cells of mice by protransfersome gel formulation used for management of
cutaneous squamous cell carcinoma. Acta Pharm.

[B20] Hartree EF (1972). Determination of protein: A modification of the Lowry method that
gives a linear photometric response. Anal Biochem.

[B21] Hornberg JJ, Laursen M, Brenden N, Persson M, Thougaard AV, Toff DB, Mow T (2014). Exploratory toxicology as an integrated part of drug discovery. Part
I: Why and how. Drug Discov Today.

[B22] Jiang M, Dong Z (2008). Regulation and pathological role of p53 in cisplatin
nephrotoxicity. J Pharmacol Exp Ther.

[B23] Kaur G, Tirkey N, Bharrhan S, Chanana V, Rishi P, Chopra K (2006). Inhibition of oxidative stress and cytokine activity by curcumin in
amelioration of endotoxin-induced experimental hepatoxicity in
rodents. Clin Exp Immunol.

[B24] Livak KJ, Schmittgen TD (2001). Analysis of relative gene expression data using real-time quantitative
PCR and the 2(-Delta Delta C(T)) method. Methods.

[B25] Mendonça LM, Dos Santos GC, Antonucci GA, Dos Santos AC, Bianchi ML, Antunes LM (2009). Evaluation of the cytotoxicity and genotoxicity of curcumin in PC12
cells. Mutat Res.

[B26] Naik RS, Mujumdar AM, Ghaskadbi S (2004). Protection of liver cells from ethanol cytotoxicity by curcumin in
liver slice culture in vitro. J Ethnopharmacol.

[B27] Nair VD, Yuen T, Olanow CW, Sealfon SC (2004). Early single cell bifurcation of pro- and antiapoptotic states during
oxidative stress. J Biol Chem.

[B28] Nesslany F, Zennouche N, Simar-Meintieres S, Talahari I, Nkili-Mboui EN, Marzin D (2007). I*n vivo* Comet assay on isolated kidney cells to
distinguish genotoxic carcinogens from epigenetic carcinogens or cytotoxic
compounds. Mutat Res.

[B29] Ognjanovic BI, Djordjevic NZ, Matic MM, Obradovic JM, Mladenovic JM, Stajin AS, Saicic ZS (2012). Lipid peroxidative damage on Cisplatin exposure and alterations in
antioxidant defense system in rat kidneys: A possible protective effect of
selenium. Int J Mol Sci.

[B30] Olfert ED, McWilliam AA (1993). Guide to the care and use of experimental animals.

[B31] Premkumar K, Kavitha S, Santhiya ST, Ramesh AR, Suwanteerangkul J (2004). Interactive effects of saffron with garlic and curcumin against
cyclophosphamide induced genotoxicity in mice. Asia Pac J Clin Nutr.

[B32] Schmid W (1975). The micronucleus test. Mutat Res.

[B33] Sedlak J, Lindsay RH (1968). Estimation of total, protein-bound, and nonprotein sulfhydryl groups
in tissue with Ellman’s reagent. Anal Biochem.

[B34] Seo SW, Han HK, Chun MK, Choi HK (2012). Preparation and pharmacokinetic evaluation of curcumin solid
dispersion using Solutol(R) HS15 as a carrier. Int J Pharm.

[B35] Serpeloni JM, Batista BL, Angeli JP, Barcelos GR, Bianchi Mde L, Barbosa FJ, Antunes LM (2013). Antigenotoxic properties of chlorophyll b against cisplatin-induced
DNA damage and its relationship with distribution of platinum and magnesium in
vivo. J Toxicol Environ Health.

[B36] Singh NP, McCoy MT, Tice RR, Schneider EL (1988). A simple technique for quantitation of low levels of DNA damage in
individual cells. Exp Cell Res.

[B37] Singh U, Barik A, Singh BG, Priyadarsini KI (2011). Reactions of reactive oxygen species (ROS) with curcumin analogues:
Structure-activity relationship. Free Radic Res.

[B38] Somparn P, Phisalaphong C, Nakornchai S, Unchern S, Morales NP (2007). Comparative antioxidant activities of curcumin and its demethoxy and
hydrogenated derivatives. Biol Pharm Bull.

[B39] Sreejayan, Rao MN (1994). Curcuminoids as potent inhibitors of lipid
peroxidation. J Pharm Pharmacol.

[B40] Stang A, Witte I (2009). Performance of the comet assay in a high-throughput
version. Mutat Res.

[B41] Swamy AV, Gulliaya S, Thippeswamy A, Koti BC, Manjula DV (2012). Cardioprotective effect of curcumin against doxorubicin-induced
myocardial toxicity in albino rats. Indian J Pharmacol.

[B42] Tice RR, Agurell E, Anderson D, Burlinson B, Hartmann A, Kobayashi H, Miyamae Y, Rojas E, Ryu JC, Sasaki YF (2000). Single cell gel/comet assay: Guidelines for in vitro and in vivo
genetic toxicology testing. Environ Mol Mutagen.

[B43] Vasconcelos T, Sarmento B, Costa P (2007). Solid dispersions as strategy to improve oral bioavailability of poor
water soluble drugs. Drug Discov Today.

[B44] Wahlstrom B, Blennow G (1978). A study on the fate of curcumin in the rat. Acta Pharmacol Toxicol.

[B45] Wolfsegger MJ, Jaki T, Dietrich B, Kunzler JA, Barker K (2009). A note on statistical analysis of organ weights in non-clinical
toxicological studies. Toxicol Appl Pharmacol.

[B46] Yallapu MM, Jaggi M, Chauhan SC (2012). Curcumin nanoformulations: A future nanomedicine for
cancer. Drug Discov Today.

[B47] Yu WG, Xu G, Ren GJ, Xu X, Yuan HQ, Qi XL, Tian KL (2011). Preventive action of curcumin in experimental acute pancreatitis in
mouse. Indian J Med Res.

[B48] Yuan JM, Li XD, Liu ZY, Hou GQ, Kang JH, Huang DY, Du SX (2011). Cisplatin induces apoptosis via upregulating Wrap53 in U-2OS
osteosarcoma cells. Asian Pac J Cancer Prev.

[B49] Zhang JG, Viale M, Esposito M, Lindup WE (1999). Tiopronin protects against the nephrotoxicity of cisplatin in the
rat. Hum Exp Toxicol.

[B50] Zhou H, Beevers CS, Huang S (2011). The targets of curcumin. Curr Drug Targets.

